# Multi-modal recommender system for predicting project manager performance within a competency-based framework

**DOI:** 10.3389/fdata.2024.1295009

**Published:** 2024-05-09

**Authors:** Imene Jemal, Wilfried Armand Naoussi Sijou, Belkacem Chikhaoui

**Affiliations:** Applied Artificial Intelligence Institute, TELUQ University, Montreal, QC, Canada

**Keywords:** recommender system, multi-modal data, natural language processing, competency-based assessment, score prediction

## Abstract

The evaluation of performance using competencies within a structured framework holds significant importance across various professional domains, particularly in roles like project manager. Typically, this assessment process, overseen by senior evaluators, involves scoring competencies based on data gathered from interviews, completed forms, and evaluation programs. However, this task is tedious and time-consuming, and requires the expertise of qualified professionals. Moreover, it is compounded by the inconsistent scoring biases introduced by different evaluators. In this paper, we propose a novel approach to automatically predict competency scores, thereby facilitating the assessment of project managers' performance. Initially, we performed data fusion to compile a comprehensive dataset from various sources and modalities, including demographic data, profile-related data, and historical competency assessments. Subsequently, NLP techniques were used to pre-process text data. Finally, recommender systems were explored to predict competency scores. We compared four different recommender system approaches: content-based filtering, demographic filtering, collaborative filtering, and hybrid filtering. Using assessment data collected from 38 project managers, encompassing scores across 67 different competencies, we evaluated the performance of each approach. Notably, the content-based approach yielded promising results, achieving a precision rate of 81.03%. Furthermore, we addressed the challenge of cold-starting, which in our context involves predicting scores for either a new project manager lacking competency data or a newly introduced competency without historical records. Our analysis revealed that demographic filtering achieved an average precision of 54.05% when dealing with new project managers. In contrast, content-based filtering exhibited remarkable performance, achieving a precision of 85.79% in predicting scores for new competencies. These findings underscore the potential of recommender systems in competency assessment, thereby facilitating more effective performance evaluation process.

## 1 Introduction

Competency-based framework for performance assessment has become highly noticeable in both academia and industry. Within the realm of education, the evaluation of competencies serves to enhance educational programs and curricula, ensuring their alignment with the evolving demands of the job market (Holmboe et al., 2010; Roegiers, 2016). On the other hand, within the job market context, competency-based frameworks serve to bridge opportunities for individuals and optimize resource allocation for employers. Moreover, they play a key role in ameliorating imbalances between supply and demand within the job market (Danielle et al., 2020). The significance of competency frameworks extends to various applications, including fostering university-industry cooperation (Kusmin et al., 2018), personalized learning environments (Paquette et al., 2021) and notably, in project management (Frederico, 2021). Esteemed organizations like the International Project Management Association (IPMA), the Project Management Institute (PMI), and the Australian Institute of Project Management (AIPM) provide tailored competency frameworks for project manager role. Furthermore, the literature presents a range of specific competency-based frameworks tailored to various domains (Shenhar et al., 2005; Takey and Carvalho, 2015; Frederico, 2021).

Designing competency-based frameworks holds a significant importance, especially in the context of employment, cannot be overstated. Research has established a strong correlation between the evaluation of competencies and employee performance (Aima et al., 2017). However, while considerable efforts have been invested in selecting competencies and establishing the framework, comparatively less attention has been paid to the evaluation process itself. This needs a closer examination, as the effectiveness of competency-based frameworks relies heavily on the evaluation methods used. Typically, competency assessment can be conducted through various means, including interviews, observation, and evidence review (Sitohang et al., 2020). Several studies have defined instruments such as the McBer Competency Framework, the Myers-Briggs Type Indicator (MBTI), the Multifactor Leadership Questionnaire (MLQ; Bass and Avolio, 1996), the Inwald Personality Inventory (IPI; Gardner, 1998), the Mayer-Salovey-Caruso Emotional Intelligence Test (Mayer et al., 2002), the Leadership Dimensions Questionnaire (LDQ; Dulewicz and Higgs, 2004), and the General Mental Ability (GMA) assessment (Schmidt and Hunter, 2004) for assessing project manager competencies. However, manually evaluating competency scores within a framework can prove to be laborious, particularly when numerous competencies are involved. This process is time-intensive, requiring the consideration of multiple evidences and artifacts, often requires the assertion of an expert. Moreover, inconsistent scoring biases among different experts pose a significant challenge.

To address these issues, we propose a solution based on a recommender system to automatically predict competency scores. This approach aims to facilitate and enhance the accuracy of competency assessment within the framework. First, we collected multi-modal data from various sources, including forms, interviews and assessment records, which were then integrated to form a comprehensive dataset. Text data have been pre-processed using natural language techniques (NLP) in order to provide a suitable data representation for the recommender system. Subsequently, we examined four recommender system approaches, including demographic, content-based and collaborative filtering, as well as hybrid approaches.

Our collaboration with PMGS, a globally recognized company in project management training and consulting, has played a crucial role in progressing this research. PMGS (PMGS Inc, 2021) has taken the lead in developing competency-based framework used for capability assessment and instructional content alignment, drawing insights from reputable sources such as IEEE standards (Recommended Practice for Defining Competencies; IEEE, 2022), the PMBOK guide published by Project Management Institute (2023) and 1EdTech (2023). This framework serves as a robust guideline foundation for assessing project manager competencies. Following interviews and collecting data from participants, a dedicated team of experts rigorously assesses the learners participants' performance by according a level of proficiency (score) for each competency within the framework. This process enables to build a competency profile which provides a comprehensive understanding of individual strengths and areas for improvement.

We evaluated the proposed solution using the collected data within the PMGS framework, the results reveal a remarkable precision rate of 81.03% in predicting competency scores. This highlights the potential of our decision support system in mitigating scoring biases and improving consistency in competency assessments. In this paper, we present several significant contributions aimed at advancing the field of competency assessment:

- We propose a novel solution that uses a recommender system for automatic score prediction.
- We integrate diverse data modalities to enhance the predictive accuracy of our system.
- We investigate NLP techniques to pre-process text data and provide adequate representation for recommender systems.
- We address the challenging cold-start problem, which arises when predicting competency scores for new project managers or when introducing a new competency into the assessment framework.

The remaining of the paper is structured as follows. Section 2 details related works which set the context for our study by highlighting prior research in the field. Section 3 details the materials and methods. Section 6 outlines the experimental results and subsequent discussion. Finally, Section 7 concludes the paper.

## 2 Related work

In the field of project management, the main focus of existing literature lies in the design of competency-based frameworks for assessment. However, limited attention has been directed toward exploring the evaluation process of competencies for performance assessment. One of the pioneering studies (Dainty et al., 2005) investigates a competency-based model aimed at predicting the performance of construction project managers. In this study, the authors identified numerous criteria defining effective performance. Data were gathered through behavioral event interviews, and logistic regression was employed for analysis to identify the crucial criteria for job performance. While this study successfully identified key criteria for effective performance, it is noteworthy that the process of identifying criteria relied on expert panel opinions, potentially introducing bias from their perspectives and possibly lacking representation of diverse viewpoints within the field. A recent (De Rezende and Blackwell, 2019) study propose a fuzzy logic model to evaluate competencies tailored specifically for companies operating within the Industry 4.0 framework. This model is designed to provide a comprehensive assessment of capabilities, with a particular emphasis on their alignment with strategic objectives.

More attention has been given to assessing competencies in the education domain. A notable contribution by Thai-Nghe et al. (2010) involves developing a recommender system tailored to predict student performance, which is closely aligned with the scope of our own research. Unlike typical recommender systems that suggest items based on predicted ratings, this system utilizes binary values (0 or 1) to represent the outcome of the initial correct attempt by students. Moreover, the authors conducted a comparative analysis between their recommender system and traditional regression methods, such as logistic and linear regression, demonstrating that the recommender system yields superior performance compared to conventional approaches. In the same vein, other studies have focused into predicting student performance using various approaches. For instance, one study (Hwang and Su, 2015) employed Clustering Locality Preserving Matrix Factorization for Student Performance Prediction, emphasizing the utilization of advanced clustering techniques for enhanced predictive accuracy. Another investigation (Xu and Yang, 2016) adopted a two-step classification approach, comprising motivation classification and grade classification, to identify distinct learner motivations and subsequently predict their likelihood of certification attainment. Additionally, a separate study (Yang et al., 2017) introduced a novel method for predicting the progression of a student's grade in massive open online courses, highlighting the ongoing innovation in predictive modeling techniques within educational research. These studies proposed interesting approaches but are applied only in education field.

The implementation of recommender systems for competency management has found diverse applications in both educational and work-related contexts. Recommender systems within the context of learning were utilized for resource recommendations, such as learning objects, courses, and genral resources (De Medio et al., 2020; Nabizadeh et al., 2020; Safarov et al., 2023). They were also employed for course recommendations relying on student-related data, as well as for suggesting competencies to improve, learning goals or outcomes (Yago et al., 2018). For instance, authors in Isaias et al. (2010) focus on managing competencies by proposing a recommender system designed to enhance the task assignment process for human resources. The system purpose is to aid the human resources department in efficiently addressing personnel needs from various departments. Similarly, Colomo-Palacios et al. (2012) introduced a hybrid recommender system that uses fuzzy logic to assist project leaders in managing software development projects, particularly within Scrum environments, by aiding in team formation for different work packages. Moreover, Guyard and Deriaz (2023) proposed a recommendation system to offer accurate profile suggestions for candidates, aligning with their competencies to fulfill job requirements.

In summary, the majority of work in project management has focused on designing a competency-based framework, while utilizing a manual procedure to evaluate competencies. Although Dainty et al. (2005) and De Rezende and Blackwell (2019) proposed automatic approaches to evaluate competencies, they used only traditional methods such as logistic regression and fuzzy logic. While some interesting methods have been used in the education field, none have been replicated in project management. Our approach differs by not only employing a well-designed framework but also introducing a new method to automatically evaluating competencies using a recommender system. Diverging from the existing literature, where recommender system techniques are commonly used to suggest materials or resources, our study utilizes a recommender system to predict scores for assessing project manager competencies. Another significant advantage of our proposed solution is the capability to integrate diverse data sources, encompassing various data modalities, including text data. We contribute to the field by utilizing NLP techniques to leverage text data in the recommender system and to enhance its capabilities. To the best of our knowledge, this study presents a pioneering multi-modal recommender system specifically designed for competency score prediction.

## 3 Materials and methods

In this section, we will describe the problem definition, outline the proposed approach for competency score prediction, and provide details regarding the multi-modal data fusion and representation as well as the recommender system approaches.

### 3.1 Problem definition

A recommender system is commonly used to suggest items for users based on their preferences and ratings. However, in this study, we adopt a recommender system to predict competencies scores for each participant where participant refers to the user, competencies refers to items and the assigned competency scores serve as ratings. Let *L* = *l*_1_, *l*_2_, …*l*_*N*_ represents the set of *N* learners, and *C* = *c*_1_, *c*_2_, …*c*_*M*_ denotes the set of *M* competencies. For each learner *l*_*i*_, an expert assigns a score for each competency *c*_*j*_, which takes discrete value within the range of [0,4]. Let *S*∈ℝ^*N*×*M*^ denotes the scoring matrix, known as the user-item matrix, where *s*_*i, j*_ represents the score of learner *l*_*i*_ on competency *c*_*j*_.

Given a dataset containing historical scores assigned to different learners across various competencies and their profiles, the goal is to predict new scores *s*_*i, j*_. As competency scores are inherently discrete values within the range of [0, 4], the predicted score is rounded to return a discrete value using a threshold of 0.5.

### 3.2 Proposed approach

**Figure 1:**
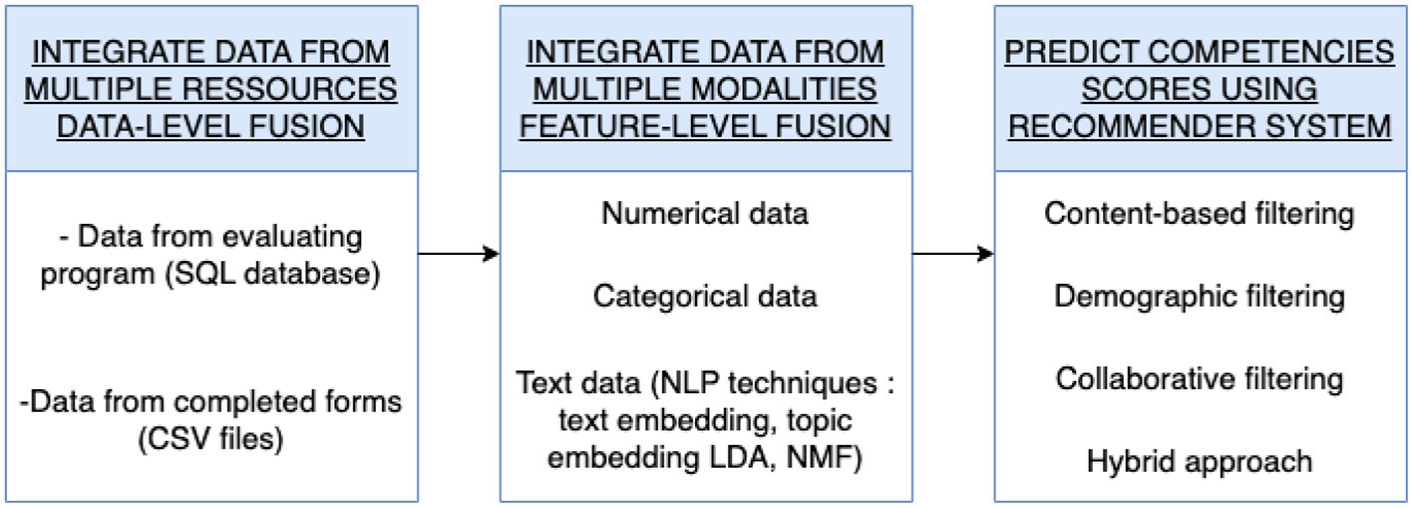
Block diagram illustrating the steps of the proposed approach.

To enhance competency score prediction in performance assessment processes, we propose a novel approach based on advanced recommender systems. The schematic representation of our methodology is illustrated in Figure 1. The process begins with data integration from diverse sources. Subsequently, we integrated data from various modalities, including numerical, categorical, and textual data. We utilize Natural Language Processing (NLP) techniques such as text embedding and topic embedding for pre-processing textual data. Competency scores are then predicted using various recommender system approaches. The first approach is content -based filtering, which predicts the score of a participant relying only on his past results. The second approach is demographic filtering, which predicts the score based on scores of participants with similar demographic characteristics. The third approach is collaborative filtering, which predicts scores based on the scoring user-item matrix S. Finally, the fourth approach combine previous techniques to predict the score.

### 3.3 Multi-modal data fusion and representation

Multi-modal data fusion is a fundamental technique in data mining that combines data from various distributions, sources, and types into a unified representation (Bramon et al., 2011). It provides richer information than a single modality can offer by leveraging specific characteristics of each modality. There are three prevalent approaches for data fusion, each executed at distinct level within the same modality (Sharma et al., 1998).

- Data-level fusion: This level provides the most detailed information. It integrates raw data of the same type typically originating from similar modality sources.
- Feature-level fusion: It involves extracting features from each data modality (Salau and Jain, 2019), followed by the fusion of these extracted features. Feature-level fusion, in contrast to data-level fusion, retains a lower level of detailed information.
- Decision-level fusion: (also known as late fusion): It is based on merging individual mode decisions or interpretations from each data modality to reach an integrated decision.

**Figure 2:**
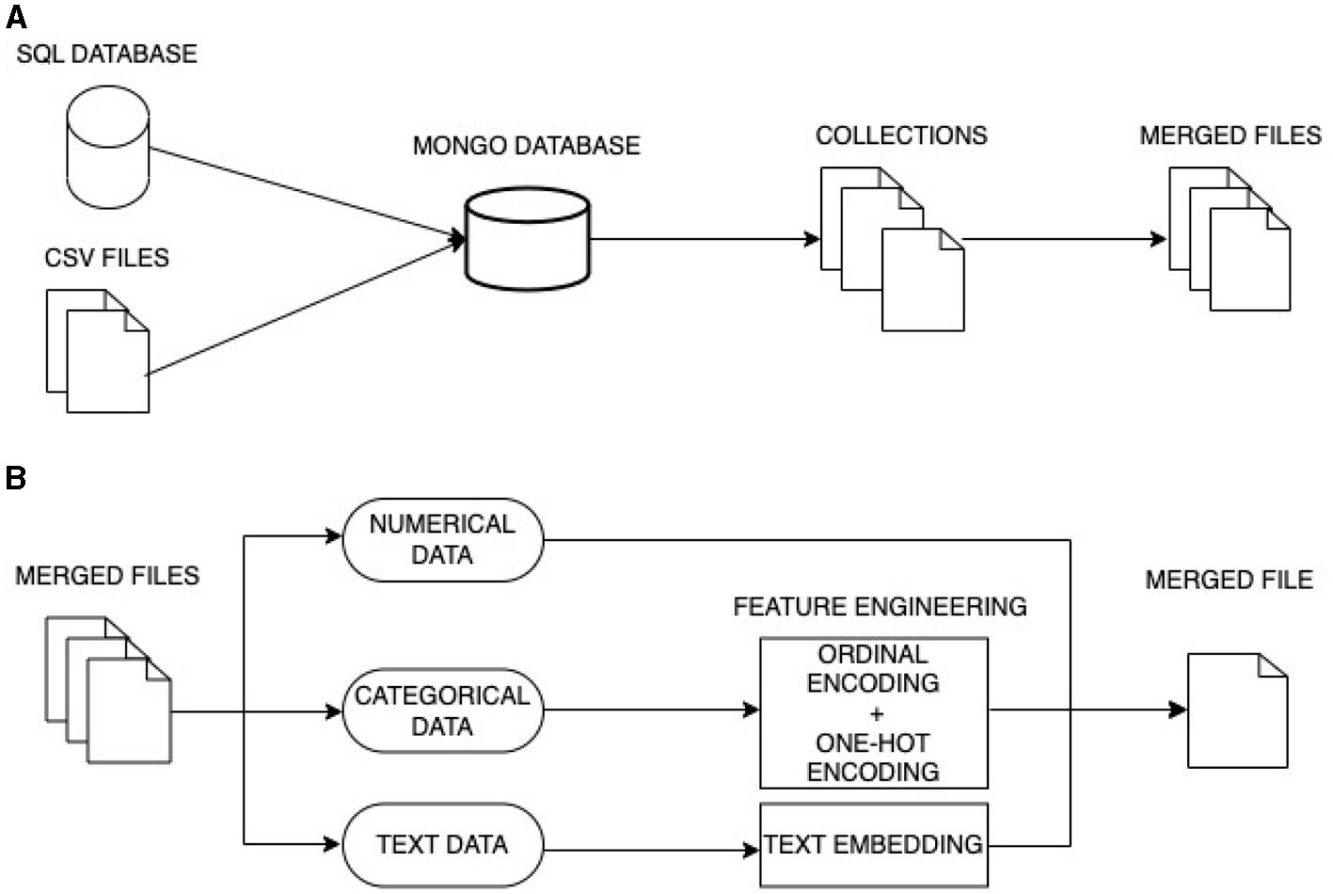
Multi-model data fusion. **(A)** First stage fusion: data-level fusion. **(B)** Second stage fusion: feature-level fusion 2.

In this study, we use data from different sources and of different types. Our data fusion process involves two main stages using data-level fusion and feature-level fusion as shown in Figure 2.

#### 3.3.1 Integrating data from multiple sources

In the first stage, we aggregate data from distinct sources which includes:

- Data obtained from an evaluating program, including interviews, stored in an SQL database.
- Data collected through completed forms, stored in CSV files.

To merge the data, we employ a Data-level fusion approach. This process begins with the transformation of SQL data into a simplified NoSQL structure, where the data is now organized into a smaller number of collections rather than being spread across numerous SQL tables. The CSV files are likewise integrated into these collections. Subsequently, these collections are merged into three separate files, each containing specific information: demographic data, details regarding competencies and achievements, and, finally, competency scores.

Utilizing NoSQL allow to handle unstructured multi-modal data such as text, images and videos coming from different sources effectively. These data need to be stored in one integrated database that facilitates access, queries, and efficient analysis. We conducted a meticulous comparison of various NoSQL datasets and ultimately opted for MongoDB as a judicious choice to store multi-modal data (Aluvalu and Jabbar, 2018). Denormalization was employed for integrating data from SQL to NoSQL.

#### 3.3.2 Integrating data from multiple modalities

The second stage employs feature-level fusion. We initiated this stage by extracting features from the various data types at our disposal:

- Numerical data: This data type is well-suited for recommender systems. The data is seamlessly integrated. It includes variables such as years of experience, the number of managed projects, and self-assessment scores.
- Categorical data: These data represent categories or groups and typically possess qualitative attributes, such as language, group names, or managed project categories. To extract features from categorical data, we employed encoding techniques like ordinal (label) encoding and One-Hot encoding.
- Text data: This data presented a unique challenge due to its complexity. To translate text data into numerical representations, we employed text embedding. This method transformed text into high-dimensional vectors, effectively capturing underlying meanings.

#### 3.3.3 Text data representation

To encode text data and extract numerical feature suitable for the recommender system, we explored two distinct Natural Language Processing (NLP) approaches : text embedding and topic modeling.

**Figure 3:**
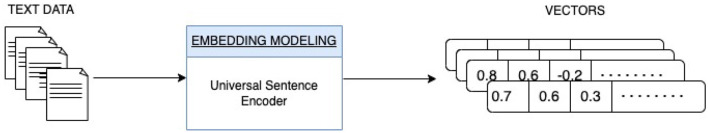
Embedding modeling for text data representation.

In the context of NLP, text embedding refers to the process of mapping text data, which is inherently high-dimensional and discrete, into a continuous, lower-dimensional vector space, while preserving the semantic and contextual information of the text (Figure 3). These vector representations, often of fixed length, are designed to capture meaningful relationships between words, phrases, sentences, or documents.

In this work we used the Universal Sentence Encoder (USE; Cer et al., 2018) to translate text data into numerical representations. The USE is a deep learning-based model designed for the embedding and representation of sentences, phrases, or short paragraphs. It is pre-trained on a large corpus of diverse textual data, allowing to provides a universal and efficient way to represent textual content, facilitating the semantic understanding and processing of sentences or short texts.

**Figure 4:**
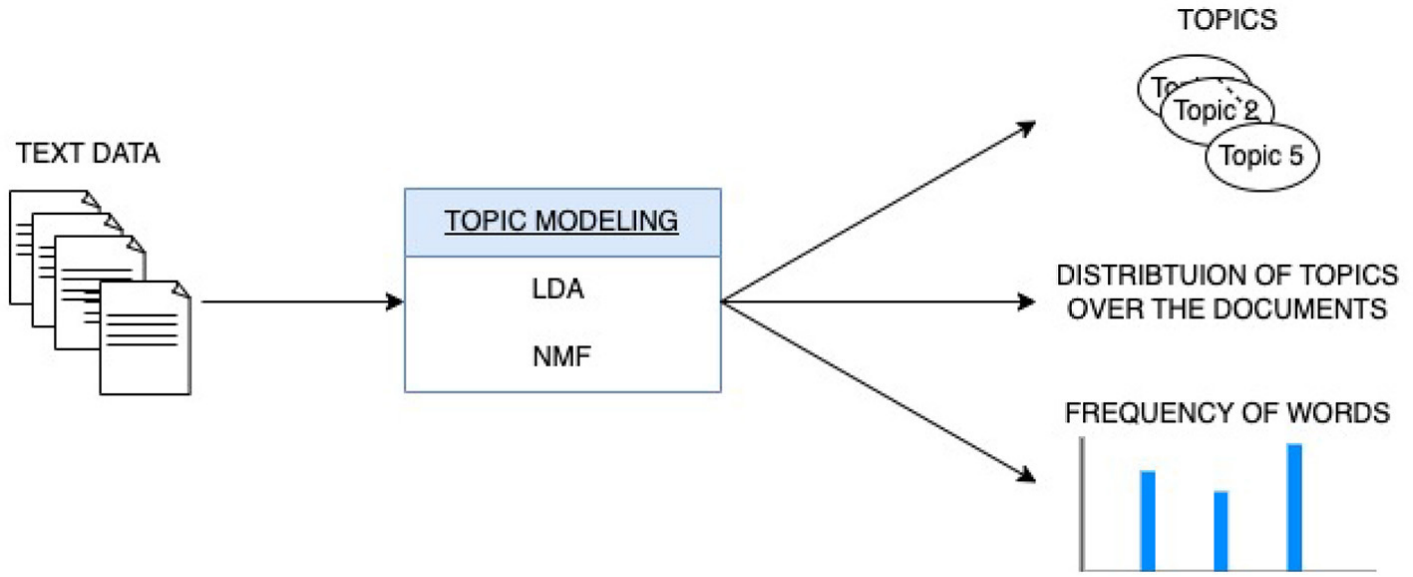
Topic modeling for text data representation.

On the other hand, topic modeling is a probabilistic statistical technique in NLP used for discovering abstract topics within a collection of documents (Figure 4). It operates by identifying patterns in the distribution of words across documents and aims to represent each document as a mixture of topics, where each topic is characterized by a distribution of words. The primary objective of topic modeling is to uncover latent semantic structures in the text data, enabling the automatic categorization and interpretation of documents based on their underlying themes.

In this work, we used two methods : the Latent Dirichlet Allocation (LDA) and Non-Negative Matrix Factorization (NMF).

- LDA is a generative probabilistic model for topic modeling in text data (Blei et al., 2003). LDA assumes that documents are mixtures of topics, and topics are mixtures of words. The model infers these topic distributions by iteratively estimating the probabilities of words occurring within topics and topics occurring within documents. It allows it to capture the hidden thematic structures in a collection of documents and assign topics to documents based on the distribution of words.
- NMF is a non-probabilistic algorithm that decomposes data using matrix factorization specifically on Term Frequency-Inverse Document Frequency (TF-IDF) transformed data (Lee and Seung, 1999). TF-IDF is a numerical statistic that measures the importance of a word in a document relative to a collection of documents, considering both the word's frequency in the document. It splits a matrix into two lower-ranked matrices. These matrices are adjusted iteratively. All entries in both matrices must be non-negative for meaningful topic interpretation.

We initiated the process with text pre-processing, which involves converting the text to lowercase, removing punctuation, and eliminating stop words. Moreover, we incorporated lemmatization, which allows reducing words to their base or root forms. Following the pre-processing phase, we applied the LDA model or the NMF model to uncover underlying topics within the text data, calculating the topic probabilities for each text. Subsequently, we determined the most probable topic for each text and assigned it accordingly. These assigned topics serve as the extracted features form the textual data.

### 3.4 Recommender systems

Recommender systems are widely used in various applications to offer personalized recommendations to users. There are different approaches of recommender systems, each with its own way of analyzing the provided data. In the following section, we will discuss the various approaches of recommender systems.

#### 3.4.1 Content-based filtering

This approach relies on metadata or features of items to make recommendations. It analyzes the characteristics of items, such as genre, color, or user profiles, to match users with similar items. By understanding the content of items and the preferences of users, content-based systems can provide recommendations that align with users' interests.

#### 3.4.2 Demographic filtering

Demographic recommender systems consider information, such as age, gender, or location, to make recommendations. This approach uses demographic data to target recommendations to users based on their shared demographic traits. Demographic information can provide valuable insights into users' preferences and help in delivering personalized recommendations.

#### 3.4.3 Collaborative filtering

Collaborative filtering is a popular approach in recommender systems that relies on the assumption that users who have shown similar preferences or behavior in the past will likely have similar preferences in the future. There are different techniques within collaborative filtering: the user-based, item-based and matrix factorization. The user-based collaborative filtering compares the preferences of a target user with other users to find similar users. It then recommends items that similar users have liked or rated positively. This technique leverages the collective wisdom of similar users to make recommendations.

On the other hand, the Item-based collaborative filtering focuses on the similarity between items. It identifies items that are similar based on user preferences and recommends items that are like the ones the user has already liked or interacted with. This technique is based on the idea that if a user likes one item, they are likely to enjoy similar items.

Finally, the matrix factorization is a class of collaborative filtering algorithms that aims to factorize a user-item interaction matrix into lower-dimensional matrices. This technique reduces the dimensionality of the data and captures latent factors or features that represent user preferences and item characteristics. Matrix factorization methods, such as singular value decomposition (SVD), non-negative matrix factorization (NMF), and deep-learning embedding factorization, are commonly used in recommender systems.

#### 3.4.4 Hybrid approach

Hybrid recommender systems combine multiple recommendation techniques to provide more accurate and diverse recommendations. There are different types of hybrid recommender systems: Nave hybrid system combine recommendations from two or more different recommendation techniques without considering their strengths or weaknesses. It simply combines them in a straightforward manner, often by giving equal weight to each method as described in Equation 1.


(1) $$\begin{array}{l} {ŝ}_{i,j}=\frac{DF_{i,j}+CB_{i,j}+CF_{i,j}}{3} \end{array}$$

where ŝ_*i, j*_ is the final predicted score or learner *i* on competency *j*, *DF*_*i, j*_, *CB*_*i, j*_, and *CF*_*i, j*_ are the demographic filtering, the content-based filtering and the collaborative filtering predicted scores, respectively.

Improved weighted hybrid systems consider the strengths and weaknesses of different techniques and use them in a more intelligent way. These systems may use a weighted combination of recommendations or dynamically switch between different techniques based on the user's preferences or the characteristics of the items (Chikhaoui et al., 2011). It is based on the number of user's ratings, *n*, to attribute different weights to each recommender system's predicted score. The final predicted score is calculated as described in Equation 2.


(2) $$\begin{array}{l} {ŝ}_{i,j}=\frac{\alpha DF_{i,j}+\beta CB_{i,j}+\gamma CF_{i,j}}{\alpha +\beta +\gamma } \end{array}$$

where ŝ_*i, j*_ is the final predicted score or learner *i* on competency *j*, *DF*_*i, j*_, *CB*_*i, j*_ and *CF*_*i, j*_ are the demographic filtering, the content-based filtering and the collaborative filtering predicted scores, respectively.

The weights α, β, and γ are calculated as follows :


$$\gamma =\frac{1}{1+\exp\left(-\frac{n}{2}\right)},\alpha =\beta =1-\gamma$$

## 4 Experiments

### 4.1 Data-set

**Figure 5:**
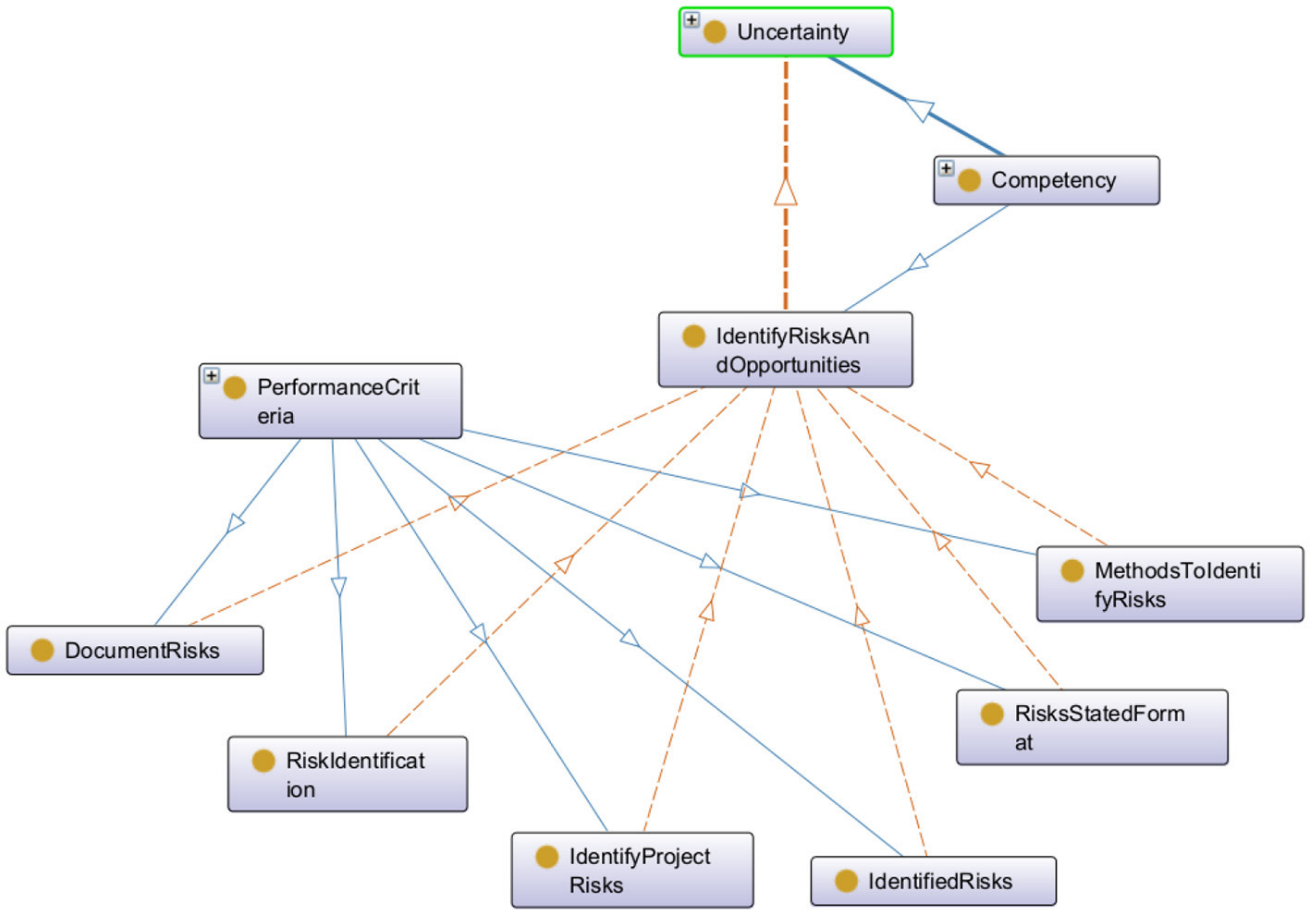
Hierarchical representation of a competency example: the competency uncertainty as a parent node with a single child which in turn having six distinct performance criteria.

The experiment was carried out using data from two groups, each containing 19 participants. These participants were subjected to interviews and survey forms for performance assessment within a competency-based framework. The framework facilitates effective evaluation and the clear identification of learning objectives and necessary training activities. Furthermore, it enhances and refines the organization of competencies by introducing a hierarchical structure ranging from the most general to the most specialized. This structure can be represented across three levels: parents or domains, children which are competencies, and grandchildren related to skills, performance criteria and knowledge. The Figure 5 below shows the hierarchy of a competency example displayed using the Protégé ontology tool.

Participants are evaluated by experts who assign an integer score between 0 and 4 to each child competency within the framework. The aim of this study is is to predict this score. The dataset contains score for 67 children's competencies per participant. Additionally, it includes demographic information such as location, job, and years of experience, as well as profile data related to achievements and competencies, such as competency names, definitions, and self-assessment scores.

### 4.2 Experimental setup

To evaluate our proposed approach for competency score prediction project management domain, we used data from 38 learners assessed across 67 competencies.

At first, we implemented 5-fold cross-validation to assess the robustness of both the content-based and demographic-based models. Subsequently, we conducted a comparative analysis of various text representation methods, including text embedding and topic modeling using LDA and NMF, with the aim of selecting the most effective method. Following this, we assessed different recommender systems, including content-based, collaborative filtering, demographic filtering, and hybrid recommender systems. Within the collaborative filtering approach, we explored multiple variations, such as item-based, user-based, embedding-based, and embedding-based with deep learning.

In the subsequent phase, we employed the leave-one-out cross-validation (LOOCV) technique to evaluate model performance in addressing the cold start problem. In LOOCV, each data point is iteratively left out of the training set, and the model is trained on the remaining data points. This process is repeated for each data point, allowing us to assess how well the model generalizes to unseen data by testing it on the data points that were left out during training. We used leave-one-learner-out cross-validation to assess the system's performance for new users and leave-one-competency-out cross-validation to evaluate its performance when applied to a new competency.

We performed several experiments to select the imputation method and the similarity metrics. Missing values were imputed using mode. Jaccard similarity was used to identify similar participants or competencies. Median was used to aggregate the scores of similar participants or competencies. We implemented the LDA algorithm with the online variational Bayes method, using the following parameters: learning-decay of 0.7, learning-offset of 10.0, a maximum of 10 iterations, and a batch size of 128. For Non-negative Matrix Factorization (NMF), we used the Coordinate Descent solver with the "frobenius" beta-loss and set a tolerance of 1e-4 for the stopping condition.

Our proposed approach is implemented in Python 3.9. The experiments utilize the TensorFlow, NLTK, and Sentence-transform packages. We employed Protégé for visualizing the framework. The experiments were conducted on a MacBook Pro 2019 with a 2.8 GHz Intel Core i7 quad-core processor and 16 GB of RAM.

### 4.3 Results and discussion

In this section, we present and discuss the results of our study on competency score prediction using a recommender system. Our findings show the effectiveness of various recommender systems in predicting scores as well as their response to the cold start problem.

#### 4.3.1 Text data representation

**Figure 6:**
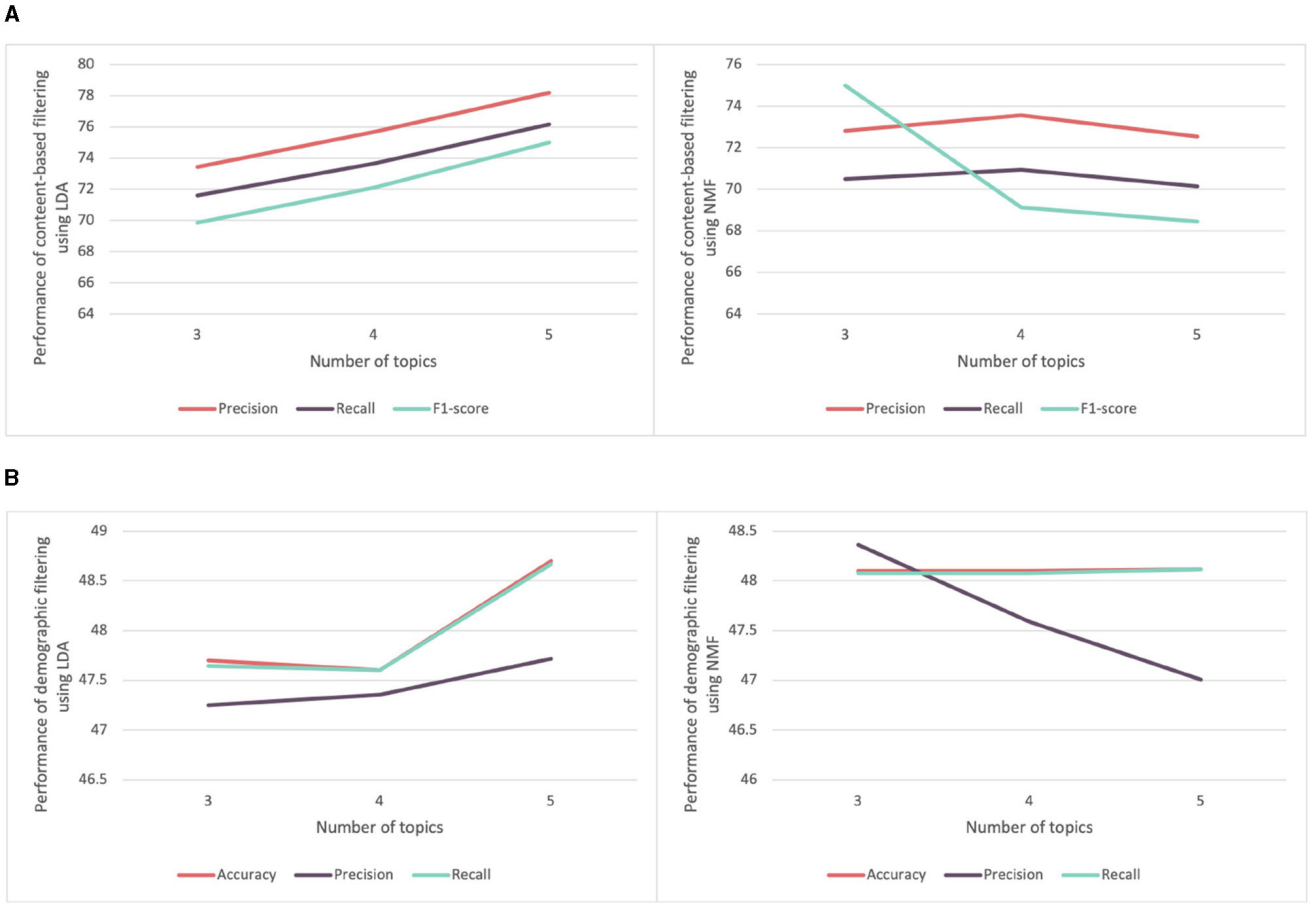
Comparison of the performance of recommender system for score prediction using features extracted from LDA and NMF. **(A)** Comparison of the performance of content-based filtering using features extracted from LDA and NMF with varying number of topics. **(B)** Comparison of the performance of the demographic filtering using features extracted from LDA and NMF with varying number of topics.

We began by evaluating content-based and demographic filtering recommender systems. Both of these systems utilize text data. In the case of demographic filtering, it involves data from participants, including paragraphs about their current and previous projects, while content-based filtering utilizes information related to competencies, such as definitions. To recall, regarding text data representation, we employed both text embedding and topic modeling using either LDA or NMF, as mentioned in Section 3.3. We conducted multiple experiments using different numbers of topics (3, 4, and 5), to evaluate both LDA and NMF methods. Figure 6A displays the performance of content-based filtering for score prediction using LDA and NMF methods to encode text data. Notably, LDA, with five topics, demonstrated the highest performance, achieving precision and recall scores of 77.75 and 75.60%, respectively. Similarly, Figure 6B illustrates the performance of demographic filtering applied to features extracted using LDA and NMF. LDA produced the best results with five topics, yielding precision, and recall values of 47.71 and 48.60%, respectively.

One illustrative example of the attributes used in topic modeling is the descriptions of current projects, where participants provide information about the projects they are managing. In Table 1, we have presented the ten keywords describing each topic, which were generated through the LDA. Each topic essentially serves as a cluster of words that tend to frequently occur within the texts. While there are variations in the specific keywords and focus within each topic, there are notable shared terms, indicating a certain degree of overlap between topics. For instance, terms like "delivery" and "project" appear across multiple topics, highlighting the recurring themes of project management and execution. Moreover, the mention of "power" in both Topics 2 and 3 signifies a connection between electrical aspects and data center projects. Additionally, Topics 3 and 5 both allude to "mv" (medium voltage), suggesting a shared emphasis on medium voltage projects. In summary, our topic modeling analysis has effectively identified five distinct topics within the data, all related to descriptions of current projects. These topics provide insights into various aspects of these projects while also highlighting certain areas of convergence.

**Table 1:** Topics identified by LDA for the current project description attribute.

**No. topic**	**Keywords**
Topic 1	Delivery, project, equinix, projects, panel, relay, protection, manufacturing, cooling, and interxion
Topic 2	Delivery, control, supply, commissioning, design, center, new, line, power, and command
Topic 3	Project, lv, data, mv, power, center, automation, digital, electrical, and scope
Topic 4	epms, delivery, phase, 15, microsoft, engineering, dub14, datacenter, 5m, and services
Topic 5	mv, eqx, project, supply, commissioning, training, design, power, manufacturing, and sat

**Figure 7:**
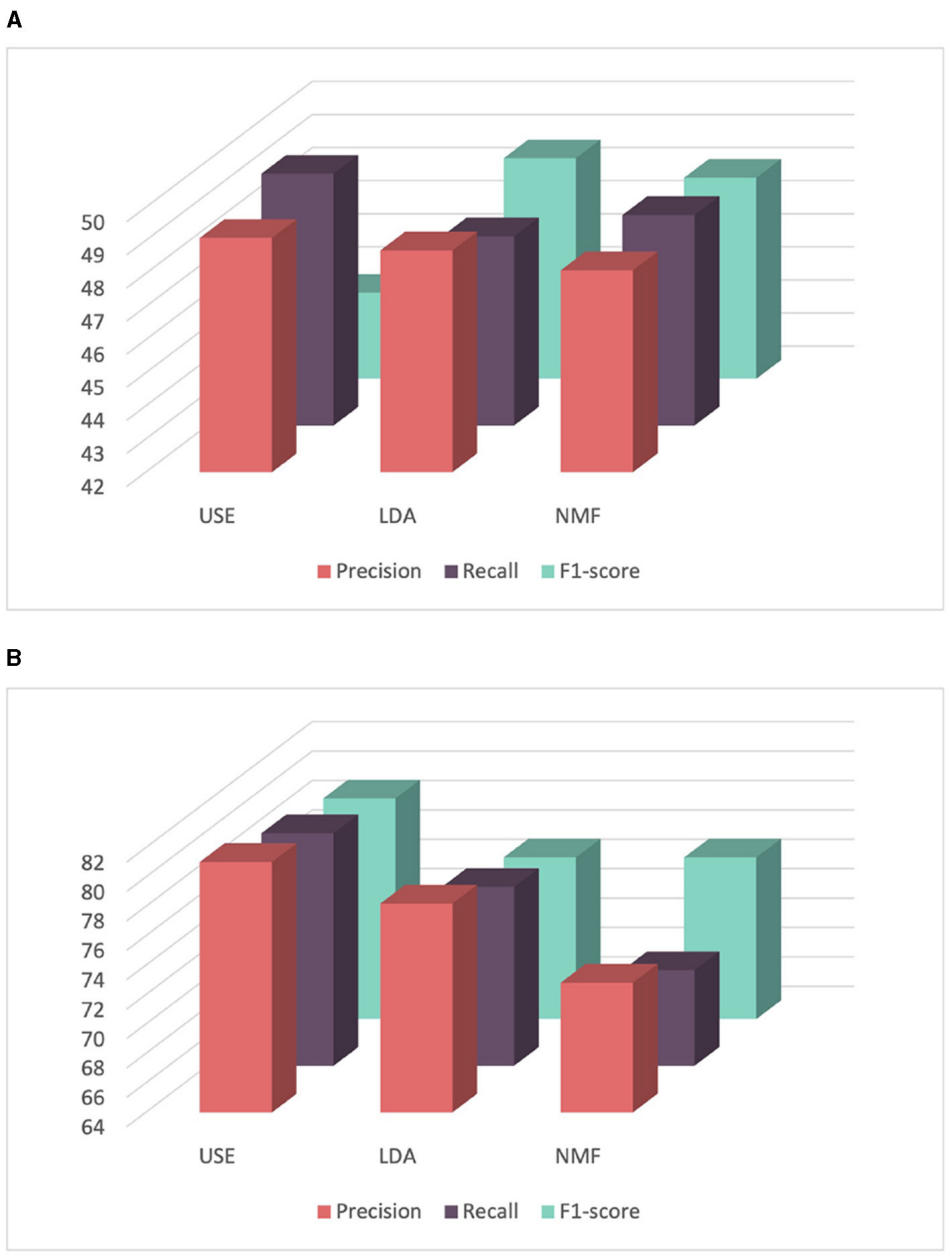
Comparison of the performance of recommender systems for score prediction using features extracted from USE, LDA, and NMF. **(A)** Comparison of the performance of content-based filtering using features extracted from USE, LDA, and NMF. **(B)** Comparison of the performance of demographic filtering using features extracted from USE, LDA, and NMF.

Finally, we explored the use of the Universal Sentence Encoder (USE) for sentence embedding. Although it expands the number of features used, it outperformed both LDA and NMF as shown in Figure 7. Content-based filtering achieved the best performance when using the USE, achieving a precision, recall and F1-score rates of 81.03, 79.81, and 79.00%, respectively. Similarly, for demographic filtering also employing USE, it achieved the highest precision, recall, and F1-score rates of 49.08, 49.61, and 44.60%, respectively. Consequently, for all recommender systems in the remaining experiments, we will opt to use the USE for text data embedding.

#### 4.3.2 Comparing recommender system performance for score prediction

**Figure 8:**
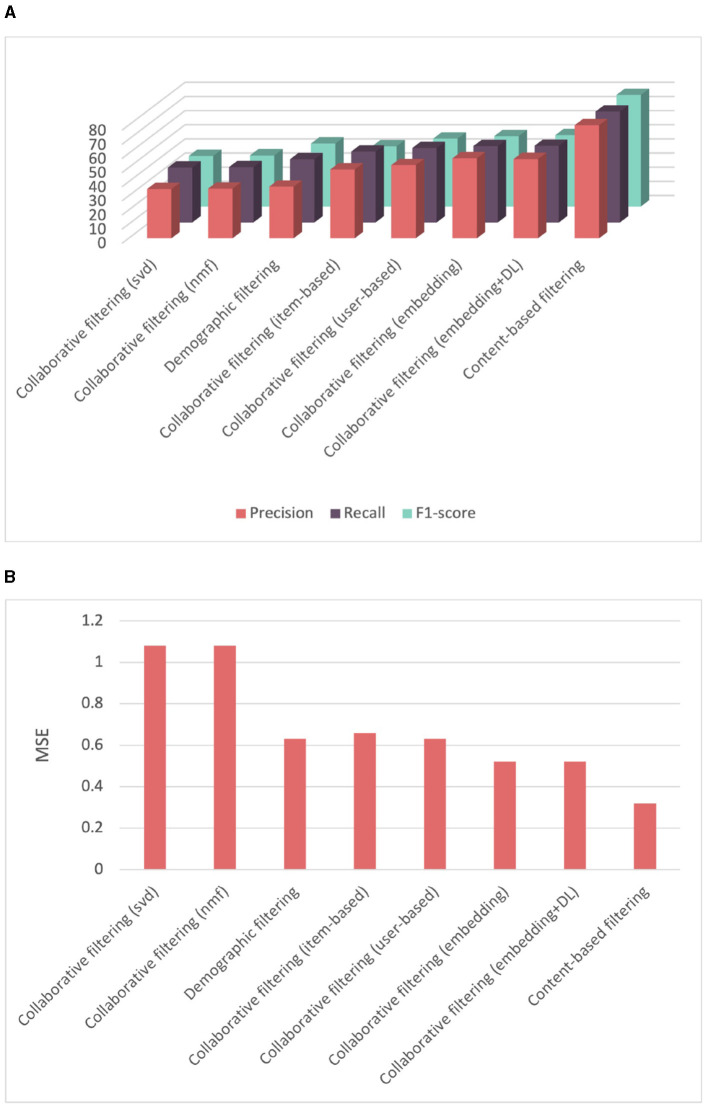
Comparison of performance among different recommender systems for score prediction evaluated using cross-validation. **(A)** Comparison of precision, recall, and F1-score among different recommender systems. **(B)** Comparison of mean squared error among different recommender systems.

To compare the performance of all recommender systems on competency score prediction, we conducted a 5-fold cross-validation and calculated the mean and standard deviation of the cross-validation scores. Table 2 provides a summary of the performance comparison of different recommender systems, including content-based, collaborative filtering, demographic filtering, and several collaborative filtering techniques such as user-based, item-based, and various implementations of matrix factorization, such as SVD, NMF, embedding, and deep learning-based embeddings. Figure 8 illustrates that the content-based recommender system demonstrates the highest performance with an averaged average precision of 81.03%, and an averaged recall of 79.81%. It is essential to highlight that the content-based approach relies on metadata associated with the items, specifically features related to competencies in our case. The framework we used incorporates a distinct hierarchy that encompass relationships among competencies, including "is child of" connections between parent and child competencies. This hierarchical structure allows us to effectively group multiple categories together. For instance, we observe that sets of child competencies under a parent competency often are similar. This can justify the good results obtained with the content-based approach. Furthermore, it is noteworthy that we can identify similarities between competencies without need to a large amount of data. In contrast, both demographic filtering and collaborative filtering failed to yield satisfactory results due to the small amount of data. The demographic filtering reached only an averaged precision and recall of 49.08 and 49.61%, respectively, with a high mean squared error of 0.63. A significant observation is that the demographic data is not well-distributed. We noticed instances where learners with higher positions lacked sufficient years of experience, yet they were entrusted with managing complex projects. This can be attributed to certain locations having a shortage of experienced project managers. For collaborative filtering, we implemented different techniques. The matrix factorization SVD and NMF collaborative filtering techniques exhibited the poorest performance, with an averaged mean squared error of 1.08. This can be attributed to the limited amount of data, which impedes the proper learning of latent features. Both user-based and item-based methods showed slightly better results but were still not satisfactory. The embedding matrix factorization and deep learning-based embedding yielded better results of 56.57 and 56.02% average precision, respectively.

**Table 2:** Evaluating recommender systems for score prediction with a cross-validation strategy.

	**Precision**	**Recall**	**F1-score**	**MSE**
Collaborative filtering (SVD)	34.81 (+/-3.16)	38.85 (+/-2.28)	35.84 +/-2.65	1.08 (+/-0.05)
Collaborative filtering (NMF)	35.13 (+/-3.33)	39.12 (+/-2.35)	36.17 +/-2.77	1.08 (+/-0.06)
Demographic filtering	49.08 (+/-1.73)	49.61 (+/-1.18)	44.60 +/-1.72	0.63 (+/-0.03)
Collaborative filtering (item-based)	48.66 (+/-2.95)	50.08 (+/-0.98)	42.66 +/-1.42	0.66 (+/-0.02)
Collaborative filtering (user-based)	51.75 (+/-2.34)	52.63 (+/-1.62)	48.04 +/-2.10	0.63 (+/-0.04)
Collaborative filtering (embedding)	56.57 (+/-3.43)	54.01 (+/-2.13)	49.82 (+/-2.74)	0.52 (+/-0.03)
Collaborative filtering (embedding+DL)	56.02 (+/-3.04)	54.17 (+/-1.93)	50.55 (+/-2.55)	0.52 (+/-0.03)
**Content-based filtering**	**81.03 (+/-1.43)**	**79.81 (+/-1.44)**	**79.00 (+/-1.70)**	**0.32 (+/-0.02)**

The best results are highlighted in bold.

**Figure 9:**
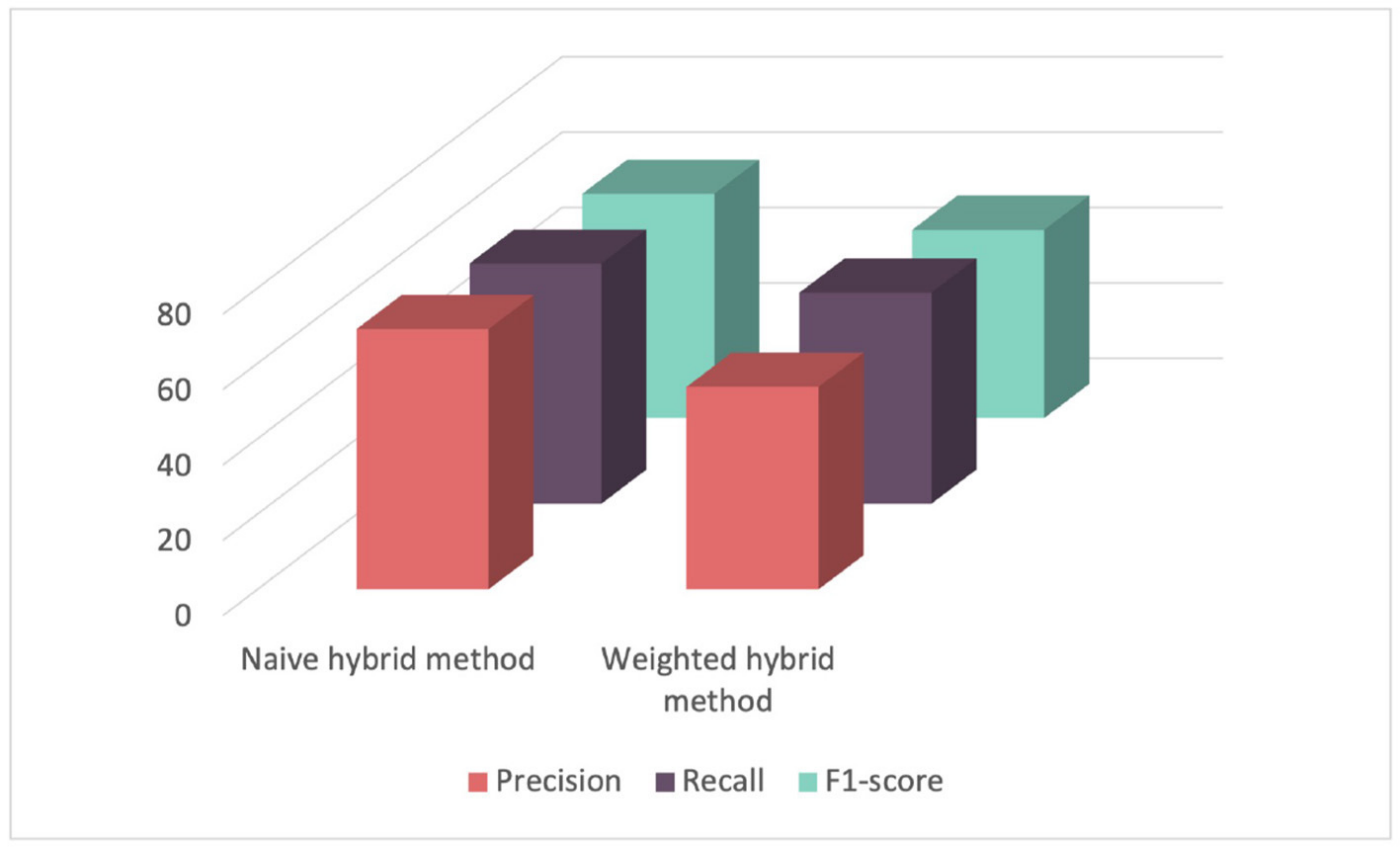
Comparison of performance among different hybrid recommender systems for score prediction evaluated using cross-validation.

In a second phase, we evaluated two hybrid recommender systems which combine content-based, demographic filtering, and collaborative filtering as mentioned in Section 3.4. The first one was a naive hybrid system where the results of the three combined recommender systems were averaged. The second one is a weighted sum of the predictions from the three predictors. As shown in Table 3 and Figure 9, the naive hybrid recommender system yielded satisfactory results, while the weighted method displayed poor results. Hence, the weighted hybrid method assigns weights based on the number of user ratings, in this case, the number of competency scores for a learner. With a small number of scores, around 50, a significant weight is given to collaborative filtering, while content-based and demographic-based methods are assigned smaller weights. This explains why the results of this approach are very similar to results obtained by the collaborative filtering recommender system. To conclude, the content-based filtering yielded the best F1-score of 79% in competency score prediction. These results are promising, especially considering that score assessment is typically conducted manually and the constraints of limited data availability. Unfortunately, the absence of comparable methods in existing literature has impeded further comparison. However, we anticipate enhancing the robustness of our analysis by incorporating additional historical data in future iterations.

**Table 3:** Evaluating hybrid recommender systems with a cross-validation strategy.

	**Precision (%)**	**Recall (%)**	**F1-score (%)**	**MSE**
Naive hybrid method	69.09 (+/-3.13)	63.75 (+/-2.29)	59.53 (+/-3.00)	0.42 (+/-0.03)
Weighted hybrid method	56.03 (+/-2.64)	53.77 (+/-1.96)	50.00 (+/-2.36)	0.53 (+/-0.03)

#### 4.3.3 Cold start problem: impact and findings

**Figure 10:**
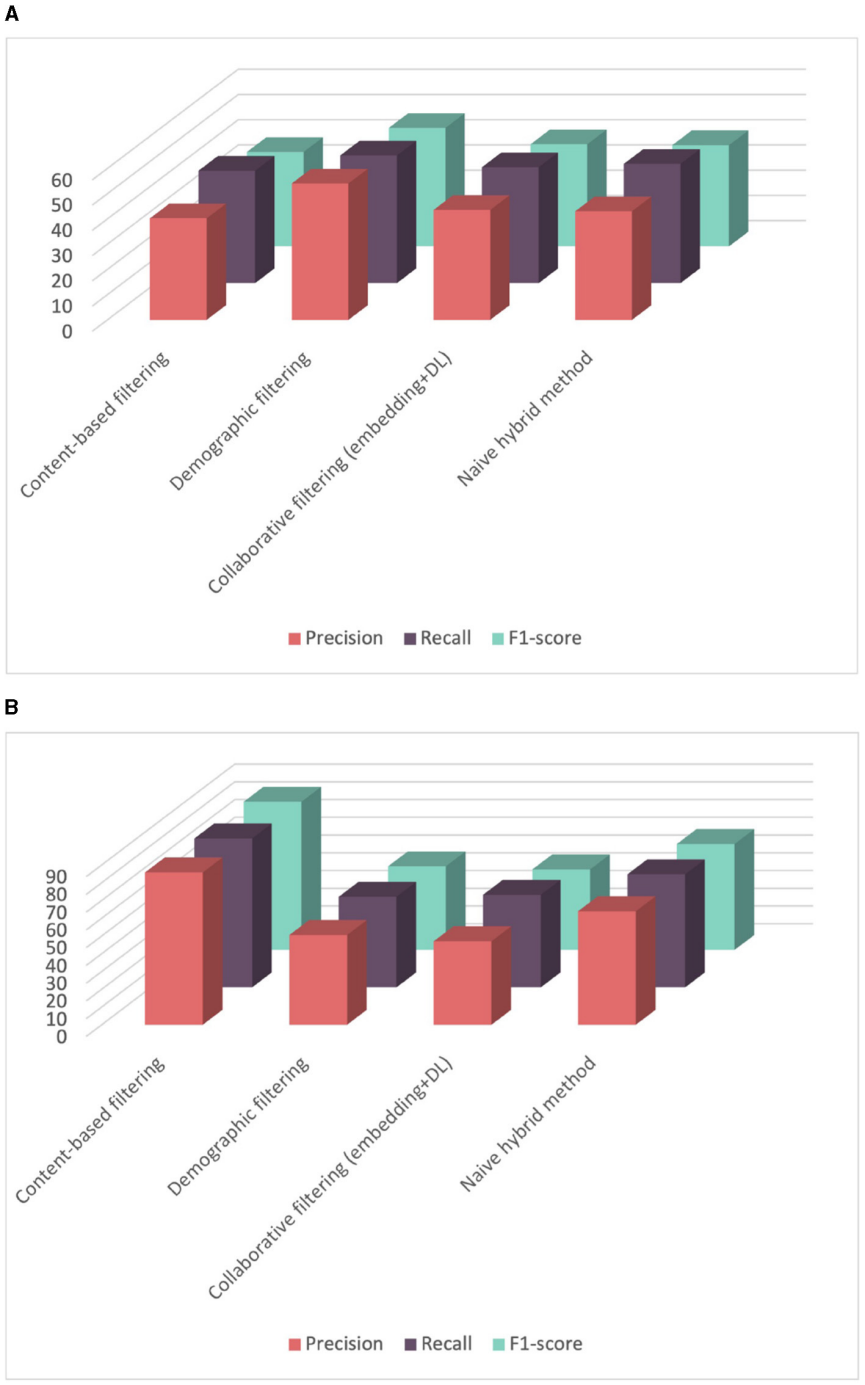
Comparison of performance among different recommender systems using leave-one-out cross-validation strategy. **(A)** Comparison of performance among different recommender systems using leave-one-learner-out cross-validation. **(B)** Comparison of performance among different recommender systems using leave-one-competency-out cross-validation.

The final step of our experiments is to evaluate how different systems respond to the cold start problem. The cold start problem in recommendation systems refers to the challenge of making accurate recommendations for new users or items that have limited or no historical data available. It occurs when the system lacks sufficient information about user preferences or item characteristics to make personalized suggestions. In our case, the challenge involves predicting competency scores for a new learner or predicting a score for a new competency. Results are illustrated in Figure 10.

Table 4 presents the averaged results obtained from evaluating different recommender systems using leave-one-learner-out cross-validation. In each experiment, we utilized data from 37 learners for training and the data from the remaining learner for testing. Notably, only the demographic filtering shows acceptable results because we only have demographic data for the new learner lacking historical score data. The average precision achieved was 54.05%, with a standard deviation of 19.12, indicating that performance depends on the similarity between the new learner and learners in the training dataset. However, the content-based filtering approach yield poor results and only achieves this performance because our scores are not balanced. The naive hybrid approach shows slight improvement over the demographic and collaborative filtering but still does not achieve satisfactory results. In summary, the performance of different approaches seems average when evaluating the approach with new users„ which inherently presents a challenging task. This outcome may be attributed to the limited dataset, as we currently only have data from 38 learners. Expanding our dataset to include a larger number of learners has the potential to significantly enhance performance.

**Table 4:** Evaluating recommender systems using leave-one-learner-out cross-validation strategy.

	**Precision (%)**	**Recall (%)**	**F1-score (%)**	**MSE**
Content-based filtering	40.23 (+/-25.37)	44.54 (+/-20.40)	37.42 +/-23.53	0.80 (+/-0.48)
**Demographic filtering**	**54.05 (+/-19.12)**	**50.59 (+/-16.29)**	**46.92 +/-19.25**	**0.62 (+/-0.25)**
Collaborative filtering	43.54 (+/-25.71)	45.95 (+/-17.66)	40.45 +/-21.51	0.71 (+/-0.39)
Naive hybrid method	43.05 (+/-24.78)	47.25 (+/-19.70)	40.10 +/-22.72	0.67 (+/-0.37)

The best results are highlighted in bold.

On the other hand, we evaluated the recommendation systems performance in predicting competency scores for newly introduced competencies through a leave-one-competency-out cross-validation strategy. This process iterates for each competency in our dataset, leaving it out for testing while using the rest for training. This process is repeated for all competencies in the dataset, allowing to assess the system performance across the entire competency spectrum. The average results across all experiments, including averaged precision, recall, F1-score, and mean squared error are displayed in Table 5. As expected, content-based filtering yields impressive results, with a precision of 85.79% and a low mean squared error of 0.28. This can be attributed to the hierarchical competency-based framework utilized, where competencies are organized into parents (domains), children (subdomains), and grandchildren (performance criteria, skills, and knowledge). Within these hierarchical structures, specific competencies display similarities. Since the content-based involves searching for comparable competencies to predict scores, this can explain the good results observed in content-based evaluations.

**Table 5:** Evaluating recommender systems using leave-one-competency-out cross-validation strategy.

	**Precision (%)**	**Recall (%)**	**F1-score (%)**	**MSE**
**Content-based filtering**	**85.79 (+/-12.94)**	**83.86 (+/-14.15)**	**83.50 +/-14.52**	**0.28 (+/-0.29)**
Demographic filtering	50.56 (+/-11.98)	51.02 (+/-9.60)	47.13 +/-10.51	0.64 (+/-0.21)
Collaborative filtering	47.04 (+/-11.57)	52.16 (+/-9.69)	45.40 +/-10.13	0.55 (+/-0.18)
Naive hybrid method	63.93 (+/-12.61)	63.67 (+/-10.83)	59.68 +/-11.80	0.45 (+/-0.21)

The best results are highlighted in bold.

On the other hand, the demographic approach depends only on imputation functions. The improvement in the naive hybrid method is mainly due to the strong results achieved by the content-based approach.

## 5 Conclusion

In this paper, we introduced a novel solution for predicting competency scores. Our solution uses a recommender system to evaluate the performance of project managers within a specific competency-based framework. A significant contribution of our work is the incorporation of multi-modal data including text data. We explored multiple recommender systems for score prediction, and content-based filtering emerged as the top-performing approach.

Moreover, we successfully addressed the cold start problem, which involves predicting competency scores for new participants or new competencies. The demographic-based system tackled the first challenge, while the content-based system addressed the latter. Our approach will serve as a decision-support system for experts, assisting them in evaluating competencies efficiently and mitigating the issue of bias between scores provided by different experts.

However, it is crucial to acknowledge a significant limitation in our study, which is the relatively small amount of data. To enhance the robustness of our recommendations, future research should consider incorporating data from other groups, which will become available shortly. Additionally, exploring alternative hybrid approaches holds promise in developing an optimal recommender system.

In this study, we focused exclusively on children competencies within the framework. In our future work, we aim to refine our solution by incorporating additional indicators of other grandchildren competencies evaluating knowledge, skills, and performance criteria. We plan to conduct additional experiments to enhance the accuracy of score prediction by incorporating a confidence level. Furthermore, we intend to address the cold start problem in score prediction for new users. Our strategy involves expanding our dataset to include more learners and enhancing the quality and diversity of content data.

These efforts will contribute to the further advance and improvement of our solution.

Finally, this solution could be extended to other competency-based frameworks, whether applied in job contexts or educational settings.

## Data availability statement

The datasets presented in this article are not readily available because, access restrictions: the dataset is restricted to authorized users only, and access is granted upon request and approval. Confidentiality: The dataset may contain sensitive or confidential information, and users are required to maintain confidentiality and data security. Requests to access the datasets should be directed to: belkacem.chikhaoui@teluq.ca.

## Author contributions

IJ: Writing – review & editing, Writing – original draft, Visualization, Validation, Software, Methodology, Investigation, Formal analysis, Data curation, Conceptualization. WN: Writing – review & editing, Writing – original draft, Visualization, Validation, Software, Methodology, Investigation, Formal analysis, Data curation, Conceptualization. BC: Writing – review & editing, Validation, Supervision, Resources, Project administration, Methodology, Funding acquisition, Formal analysis, Conceptualization.
